# Metapopulation Structure of CRISPR-Cas Immunity in *Pseudomonas aeruginosa* and Its Viruses

**DOI:** 10.1128/mSystems.00075-18

**Published:** 2018-10-23

**Authors:** Whitney E. England, Ted Kim, Rachel J. Whitaker

**Affiliations:** a Department of Microbiology, University of Illinois at Urbana-Champaign, Urbana, Illinois, USA; b Carl R. Woese Institute for Genomic Biology, University of Illinois at Urbana-Champaign, Urbana, Illinois, USA; Queen’s University Belfast

**Keywords:** CRISPR, *Pseudomonas aeruginosa*, bacteriophage evolution, cystic fibrosis, evolution, host-virus interactions, microbiome

## Abstract

Viruses that infect the widespread opportunistic pathogen Pseudomonas aeruginosa have been shown to influence physiology and critical clinical outcomes in cystic fibrosis (CF) patients. To understand how CRISPR-Cas immune interactions may contribute to the distribution and coevolution of P. aeruginosa and its viruses, we reconstructed CRISPR arrays from a highly sampled longitudinal data set from CF patients attending the Copenhagen Cystic Fibrosis Clinic in Copenhagen, Denmark (R. L. Marvig, L. M. Sommer, S. Molin, and H. K. Johansen, Nat Genet 47:57–64, 2015, https://doi.org/10.1038/ng.3148). We show that new spacers are not added to or deleted from CRISPR arrays over time within a single patient but do vary among patients in this data set. We compared assembled CRISPR arrays from this data set to CRISPR arrays extracted from 726 additional publicly available P. aeruginosa sequences to show that local diversity in this population encompasses global diversity and that there is no evidence for population structure associated with location or environment sampled. We compare over 3,000 spacers from our global data set to 98 lytic and temperate viruses and proviruses and find a subset of related temperate virus clusters frequently targeted by CRISPR spacers. Highly targeted viruses are matched by different spacers in different arrays, resulting in a pattern of distributed immunity within the global population. Understanding the multiple immune contexts that P. aeruginosa viruses face can be applied to study of P. aeruginosa gene transfer, the spread of epidemic strains in cystic fibrosis patients, and viral control of P. aeruginosa infection.

**IMPORTANCE** Pseudomonas aeruginosa is a widespread opportunistic pathogen and a major cause of morbidity and mortality in cystic fibrosis patients. Microbe-virus interactions play a critical role in shaping microbial populations, as viral infections can kill microbial populations or contribute to gene flow among microbes. Investigating how P. aeruginosa uses its CRISPR immune system to evade viral infection aids our understanding of how this organism spreads and evolves alongside its viruses in humans and the environment. Here, we identify patterns of CRISPR targeting and immunity that indicate P. aeruginosa and its viruses evolve in both a broad global population and in isolated human “islands.” These data set the stage for exploring metapopulation dynamics occurring within and between isolated “island” populations associated with CF patients, an essential step to inform future work predicting the specificity and efficacy of virus therapy and the spread of invasive viral elements and pathogenic epidemic bacterial strains.

## Abstract (precis)

Pseudomonas aeruginosa is a widespread opportunistic pathogen and a major cause of morbidity and mortality in cystic fibrosis patients. Microbe-virus interactions play a critical role in shaping microbial populations, as viral infections can kill microbial populations or contribute to gene flow among microbes. Investigating how P. aeruginosa uses its CRISPR immune system to evade viral infection aids our understanding of how this organism spreads and evolves alongside its viruses in humans and the environment. Here, we identify patterns of CRISPR targeting and immunity that indicate P. aeruginosa and its viruses evolve in both a broad global population and in isolated human “islands.” These data set the stage for exploring metapopulation dynamics occurring within and between isolated “island” populations associated with CF patients, an essential step to inform future work predicting the specificity and efficacy of virus therapy and the spread of invasive viral elements and pathogenic epidemic bacterial strains.

## INTRODUCTION

Viral infection is known to have considerable impact on the evolution of microbial communities in all environments, including the human microbiome, where viruses act both as bacterial antagonists and agents to transfer novel and important bacterial traits (1–5). Comparisons of even small numbers of Pseudomonas aeruginosa genomes have revealed a dynamic variable genome replete with horizontally transferred elements, many of which are proviruses and virus-like elements (6, 7). These viral elements contain genes critical for P. aeruginosa infection and pathogenicity; for example, the temperate cytotoxin-converting virus phiCTX encodes a toxin shown to increase P. aeruginosa virulence in a mouse model (8). Other proviruses have influenced various functions important for microbial colonization and persistence, including cell adhesion, resistance to phagocytosis, and exopolysaccharide digestion for biofilm remodeling (9). Notably, proviruses likely play an important role in the Liverpool epidemic strains (LES), which are responsible for 10% of cystic fibrosis (CF)-associated infections in the United Kingdom (10). These strains are adept at colonizing the lung, display increased antibiotic resistance, and are associated with worse clinical outcomes, including greater loss of lung function and higher rates of lung transplantation and death (11). Some colonization advantages of these strains have been shown to lie in integrated proviruses in the LES genome. These elements contain genes homologous to known P. aeruginosa viruses; prophages 2 and 3 are related to F10, prophage 4 to D3112 and DMS3, prophage 5 to D3, and prophage 6 to Pf1 (12). Disrupting three of these proviruses (prophages 2, 3, and 5) has been shown to create strains attenuated relative to the wild-type ancestor in a rat lung chronic infection model (12). Some of these integrated viruses also retain their lytic activity and may affect P. aeruginosa density in chronic CF lung infections, where they are induced by stress such as antibiotic treatment (13).

The evolution of P. aeruginosa viruses and their impacts on bacterial dynamics and fitness are shaped by CRISPR-Cas (clustered regularly interspaced short palindromic repeats) immunity (14–18). The CRISPR system is composed of two parts: CRISPR arrays of the eponymous repeats interspersed with short DNA fragments called spacers, and a set of CRISPR-associated (Cas) genes, which carry out CRISPR system functions. The sequences of spacers in these arrays come from foreign genetic elements such as viruses at matching locations in the element genome called the protospacer. New spacers are acquired and integrated from the protospacer of the virus into one end of the array, known as the leader providing the adaptive function. Arrays are transcribed from the leader end, processed into cr-RNAs containing a single spacer, and bound to the functional CRISPR-Cas complexes. When a complex containing a cr-RNA matches a protospacer in a targeted element, the element is degraded by Cas proteins, providing immunity.

P. aeruginosa is known to harbor two subtypes of the type I CRISPR system in its genome: I-E and I-F. Type I-F CRISPRs are considerably more common than type I-E, appearing in 33% of genomes versus 3% for type I-E in a study of 122 clinical isolates (19). The type I-F system has been shown to be fully functional as an immune system, conferring immunity to multiple temperate viruses and adding new spacers in response to challenge with a lytic virus (20). In addition to these genomically encoded CRISPRs, a type I-C system has been identified on an integrative and conjugative element present in some P. aeruginosa strains (21). Some common P. aeruginosa laboratory strains, including PAO1, lack CRISPRs; however, others, such as PA14, contain complete CRISPR systems. LES and related strains (22) contain a single, well-conserved type I-F array but lack associated Cas genes, suggesting an ancestral partial loss of the system rendering it nonfunctional. P. aeruginosa genomes with intact CRISPRs are smaller than CRISPR-less genomes, consistent with the CRISPR system preventing integration of viruses and mobile elements (21). Since some CRISPR-targeted elements have been connected to P. aeruginosa virulence, it has been suggested that absent or nonfunctional CRISPRs may allow strains to acquire and maintain these virulence islands (12, 19, 23).

Up to a quarter of spacers from all CRISPR subtypes have been shown to match viruses or proviruses (19, 21), indicating that P. aeruginosa has recorded numerous encounters with viruses in its CRISPR arrays. CRISPR arrays have been used as variable molecular markers to classify P. aeruginosa strains (21). Here, we compare CRISPR spacers from a spatially restricted longitudinal data set to those from the global P. aeruginosa population to determine if CRISPR diversity and interactions with viruses vary among these populations. In doing so, we contrast the local and global structure of immunity and identify highly targeted virus clusters that infect P. aeruginosa.

## RESULTS

### Within-host and between-patient CRISPR diversity.

**FIG 1:**
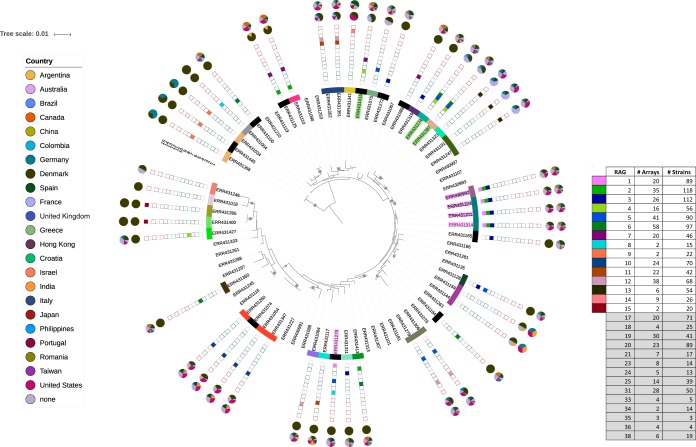
MLST tree of Copenhagen strains. Maximum likelihood tree is built from 7-locus MLST of Copenhagen strains. Strains are labeled by Sequence Read Archive accession number. Only one representative per MLST+CRISPR-type combination is shown. Bootstrap values >70 are shown as gray circles. Inner ring of colored bars represents the set of spacers in each strain; matching colors indicate completely shared spacers. Black bars represent spacer sets unique to a single strain in the entire data set. Stacked colored squares show presence (filled) or absence (open) of 15 core related array groups (RAGs). Gray-shaded table cells contain global RAGs found when comparing Copenhagen arrays to arrays from other data sets, which are not illustrated in the tree. Pie charts show counts by country of identical (inner) or related (outer) arrays in strains outside the Copenhagen data set. Related arrays are defined as those sharing two or more consecutive identical spacers. Two examples of RAG recombination (identical or related arrays in combination with unrelated arrays in different strains) are highlighted with colored boxes around strain names: RAG 1 (lavender) and RAG 4 (light green).

We assembled CRISPR spacers into arrays from a published sequence read from a longitudinal set of 458 isolates of P. aeruginosa from 34 patients at the Copenhagen Cystic Fibrosis Clinic (24) (here referred to as the Copenhagen data set; see Table S1 in the supplemental material). Isolates were derived from sputum samples from children and young adults with CF ranging in age from 1.4 to 26.3 years of age, with patients being sampled longitudinally over a period of one to ten years between 2001 and 2013 (24). For each strain we also constructed a seven-locus multilocus sequence type (MLST) (25) to represent the core genome of the strain. To account for identical clones sequenced repeatedly in the Copenhagen data set, we collapsed all strains with identical MLSTs and CRISPR sequences which originated from the same patient to a single representative, resulting in a clone-corrected data set with 72 isolates. In total we find that 46 of these 72 strains contain CRISPRs, with 83 unique CRISPR arrays (multiple arrays per strain) (Fig. 1) in the Copenhagen data set. We constructed a phylogeny based on MLST data of 72 strains and mapped CRISPR arrays onto this phylogeny (Fig. 1). We observe that CRISPR array sequences map onto the MLST phylogeny, suggesting a linked evolutionary history of CRISPR with the core genome. New CRISPR variants (marked with black boxes in Fig. 1) evolve among very closely related strains with identical MLSTs. This is consistent with previous studies showing that CRISPRs largely evolve with core gene loci but show more recent variation. Most strains in this data set have multiple CRISPR arrays, with the majority containing three or fewer arrays (Fig. S1A). Across all arrays, these strains contain 4 to 65 spacers, with an average of 35.4 spacers per strain (Fig. S1B).

10.1128/mSystems.00075-18.1 FIG S1 Distribution of CRISPR spacers in arrays in the Copenhagen CF patient population. Count of CRISPR arrays (A) and spacers (B) per strain in strains from the clone-corrected Copenhagen data set. Download FIG S1, EPS file, 0.4 MB. Copyright © 2018 England et al. 2018 England et al. This content is distributed under the terms of the Creative Commons Attribution 4.0 International license.

10.1128/mSystems.00075-18.8 TABLE S1 P. aeruginosa strains and spacer sequences used in this study. Download Table S1, XLSX file, 0.3 MB. Copyright © 2018 England et al. 2018 England et al. This content is distributed under the terms of the Creative Commons Attribution 4.0 International license.

To look for evolution in CRISPR arrays within a patient, we compared assembled arrays from longitudinal samples. Of the 34 longitudinally sampled patients with any change in the spacer content in their CRISPR arrays over time, we find this variation results from spacer deletion in only two patients, with no examples of spacer addition (Fig. S2). Most strains maintained their CRISPR arrays over time (up to 10 years, 2 months), neither deleting existing arrays nor incorporating new arrays. These data suggest that CRISPR immunity profiles change minimally within a human host over the time course of an infection. We note that in ten of these patients, new strains were identified with unrelated CRISPR spacer profiles in new clonal backgrounds (24). This data set has very few isolates from each sample (maximum of 9, average 1.6, SD ±1.1), so we cannot distinguish whether patients were already infected by multiple strains or newly colonized. Although this may change the immune profile of the within-host population, it is not evidence of active within-host CRISPR evolution.

10.1128/mSystems.00075-18.2 FIG S2 Related array groups containing patient-unique arrays. RAGs are demarcated by thick black lines. Each box in an array represents a spacer; boxes of different colors represent variant spacers. White boxes represent identical spacers shared among arrays within a RAG. The leader end of each array is to the left. Observations outside Copenhagen patients and prevalence within each Copenhagen patient (isolates with array/total isolates from patient) are shown below each array. RAGs showing evidence of within-patient deletions are marked with an asterisk. RAGs unique to the Copenhagen patient population are marked with a ‡. Download FIG S2, EPS file, 1.0 MB. Copyright © 2018 England et al. 2018 England et al. This content is distributed under the terms of the Creative Commons Attribution 4.0 International license.

To identify related CRISPR arrays in the Copenhagen population, arrays from all Copenhagen patients were grouped into sets of related arrays sharing at least two sequential spacers (related array groups, or RAGs). In total, we found 15 RAGs in the Copenhagen population (Fig. 1). Most arrays within RAGs contained deletions (24) at the trailer end of the array (i.e., L798), four contained insertions (i.e., L804), and three lacked sufficient examples to determine if the change was an insertion or deletion relative to its ancestor (Fig. S2). In addition, we observed 15 examples with differences at the leader end among RAGs from different patients. These additions occur presumably from spacer addition, ranging from one to two spacers (i.e., L356 and L795) to the majority of the array (up to 24 variant spacers in L707 and L787) (Fig. S2). This greater among-patient variation suggests that change in CRISPR arrays, including the addition of new CRISPR spacers, is occurring among but not within patients.

### Local CRISPR diversity reflects the global population.

We compared the CRISPR arrays found within the local data set to 726 publicly available P. aeruginosa sequences. These sequences come from strains isolated over 25 years from 26 countries, originating from CF, human non-CF, and environmental sources (Fig. S3). Out of a total of 1,184 P. aeruginosa strains, 754 (64%) contain known CRISPR repeats. We identified 3,152 unique spacer sequences in 729 unique arrays that differ by at least one spacer. A rarefaction curve of spacer sequences reveals that despite a broad sampling of P. aeruginosa sequence data, it is unlikely that all spacers in the population have been observed (Fig. S4). Unlike our assembly of CRISPRs from the Copenhagen data set, strains containing identical CRISPRs are not clone-corrected, as none of these strains are known to originate from the same sample or individual. However, 97 strains isolated across four continents (Europe, Asia, and North and South America), over 22 years (1990 to 2012), and in varied environments (CF, non-CF human, and environmental samples) contained arrays identical to those in the Copenhagen population (Table S1). In addition to these identical arrays, we identified 29 RAGs containing 437 arrays which have at least two consecutive spacers in common with a Copenhagen array. Three RAGs (groups 8, 9, and 15) are not observed outside the Copenhagen data set, and eight of the RAGs contained array variants unique to an individual within the Copenhagen data set (Fig. S2). RAGs across the global data set range in size from pairs to groups of over 50 (Fig. 1, RAG table), reflecting long-term CRISPR diversification in the global population. Like variation among patients, this variation was seen in deletion and addition of spacers. In total, 80 out of 87 (92%) unique arrays in the Copenhagen study were shared exactly with (30, 34.5%) or related to (76, 87.4%) arrays found outside Copenhagen. As many arrays have exact and related versions in the global population, these percentages do not equal 100. This widespread distribution of related arrays shows that the Copenhagen CF population reflects the CRISPR diversity of the global population.

10.1128/mSystems.00075-18.3 FIG S3 Isolation environment, geographical location, and year of isolation for 1,184 P. aeruginosa sequences used in this study. “Non-CF” refers to strains isolated from humans without CF. “Human” refers to strains isolated from a human whose CF status is unknown. “CF clones” denotes strains isolated from the same individual which are identical at the MLST and CRISPR level. Download FIG S3, TIF file, 0.4 MB. Copyright © 2018 England et al. 2018 England et al. This content is distributed under the terms of the Creative Commons Attribution 4.0 International license.

10.1128/mSystems.00075-18.4 FIG S4 Spacer rarefaction curve. Rarefaction curve of spacers identified in 1,184 P. aeruginosa genomes. Download FIG S4, EPS file, 0.6 MB. Copyright © 2018 England et al. 2018 England et al. This content is distributed under the terms of the Creative Commons Attribution 4.0 International license.

We also observe arrays from the same RAG appearing in the same genome as arrays of other RAGs in various combinations, indicating recombination of entire arrays between strains. We examined the number of combinations of the 29 RAGs in the Copenhagen and global data sets. Within the Copenhagen population, there are 186 strains with at least two arrays in RAGs; these include 19 unique combinations of at least two RAGs, with individual RAGs appearing in almost two different combinations on average (1.90, SD ±1.44). Two examples of this are highlighted in Fig. 1; arrays from RAGs 1 (lavender) and 4 (light green) each appear in combination with two sets of unrelated arrays. In the global data set, there are 417 strains with two or more RAGs, and we find broader variation in RAG combinations, with 68 unique combinations and RAGs appearing in nearly six combinations on average (5.83, SD ±4.68) All 19 RAG combinations from the Copenhagen population are observed, along with 49 novel global RAGs.

### Identifying virus clusters.

**FIG 2:**
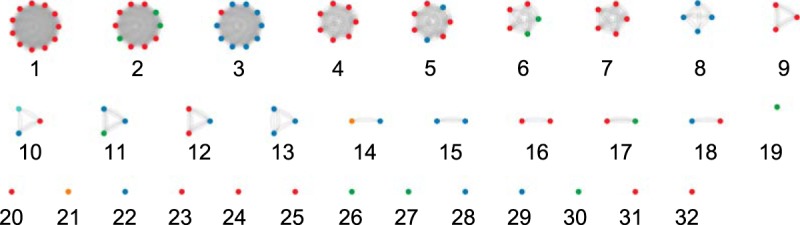
Virus genome clusters. The color of each node indicates the if the virus is lytic (red), temperate (blue), nonlytic (orange), or unknown (green). Clusters 1 to 18 contain multiple members and are connected by edges to other cluster members with which they share at least 0.2 PLA. Unclustered viruses (singletons) are numbered 19 to 32.

To understand CRISPR interactions with different virus types, we classified known P. aeruginosa viruses into clusters. We gathered 92 sequenced viruses along with six characterized proviruses integrated in the genome of epidemic strain LESB58 (12) (Table S2). We divided our virus library into clusters based on the fraction of the virus genomes aligned in pairwise BLAST searches, with a minimum of 20% aligned (see Materials and Methods). This produced 18 virus clusters with at least two members, as well as 14 singletons which did not fall into clusters (Fig. 2). These clusters are consistent with and augment previous analyses of virus families using smaller sets of well-characterized P. aeruginosa viruses (26, 27).

10.1128/mSystems.00075-18.9 TABLE S2 Virus strains used in this study. Download Table S2, XLSX file, 0.02 MB. Copyright © 2018 England et al. 2018 England et al. This content is distributed under the terms of the Creative Commons Attribution 4.0 International license.

These clusters reflect known features of P. aeruginosa viruses. Some viruses produce anti-CRISPR proteins which interfere with CRISPR immunity (28–30); all such viruses in our data set are in cluster 03, which contains largely lysogenic mu-like viruses as well as LES prophage 4. While many clusters are exclusively lytic or temperate, some, such as clusters 03, 05, and 11, contain a mixture of viral lifestyles. For example, lytic PA1/KOR/2010 has high homology to temperate members of cluster 03 but lacks a *c* repressor critical for lysogeny (31). A complete list of viruses and their assigned clusters is in Table S2.

### CRISPR matches to P. aeruginosa viruses.

**FIG 3:**
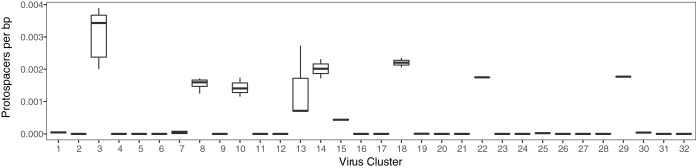
Variable targeting of virus clusters. Box plot of protospacers per base pair of viral sequence for each virus cluster. The center line of the box represents the median; the upper and lower lines mark the first and third quartiles, respectively. Whiskers extend to 1.5× the interquartile range; outliers are shown as black dots.

We compared all spacers in our library to the viral data set. In total, 1,172 spacers (37.2%) match these viruses with up to four mismatches across the length of the spacer, and with an appropriate protospacer-adjacent motif (PAM). Remarkably, 1,980 spacers (62.8%) match no viruses in this data set, indicating that CRISPRs are sampling a genome space of viruses and other elements that are not included in this data set. Of the 98 virus sequences, 46 (46.9%) contained a protospacer matched by at least one spacer. Unmatched viruses include all members of clusters 02, 04, 05, 06, 09, 11, 12, 16, and 17, along with singletons 20, 21, 23, 24, 26, 27, 31, and 32 (Fig. 3). With the exception of cluster 11, all these clusters are predominantly lytic (Fig. 2). The total number of protospacer matches per viral genome varied from 1 to 142 (Fig. S5). The clusters with the most protospacers are predominantly temperate, including proviruses (Fig. S5 and Table S2). Our data support the finding that temperate viruses contain more protospacers than viruses characterized as lytic (19) (Fig. S5, Welch’s *t* test, *P* = 6.127e−07).

10.1128/mSystems.00075-18.5 FIG S5 Spacers target temperate viruses. (A) Number of protospacers per base pair of sequence in 97 sequenced viruses categorized by lifestyle. (B) Number of protospacers per base pair is higher in temperate viruses than in lytic viruses (Welch’s *t* test, *P* = 6.127e−07). The center line of the box represents the median; the upper and lower lines mark the first and third quartiles, respectively. Whiskers extend to 1.5× the interquartile range; points outside this range are shown as black dots. Download FIG S5, EPS file, 0.4 MB. Copyright © 2018 England et al. 2018 England et al. This content is distributed under the terms of the Creative Commons Attribution 4.0 International license.

To see if protospacers were more likely to be shared between closely related strains, we compared shared protospacers in pairs of viruses to the proportion of their genomes that align (PLA, see Materials and Methods). We found a positive relationship between PLA and shared protospacers (Fig. S6C, *r* = 0.85, *P* < 2e−16), showing as expected that viruses with similar genomes share more protospacers.

10.1128/mSystems.00075-18.6 FIG S6 PLA and shared protospacers among viruses. (A) Heat map of proportion length aligned (PLA) between viral genomes. Genomes are grouped by cluster (colored bars below/left). (B) Protospacers shared between virus clusters. Heat map indicates the proportion of protospacers shared between each cluster pair. Cluster lacking protospacers are omitted. (C) Genome similarity (PLA) correlates with proportion of protospacers shared between virus strains. Download FIG S6, EPS file, 3.0 MB. Copyright © 2018 England et al. 2018 England et al. This content is distributed under the terms of the Creative Commons Attribution 4.0 International license.

**FIG 4:**
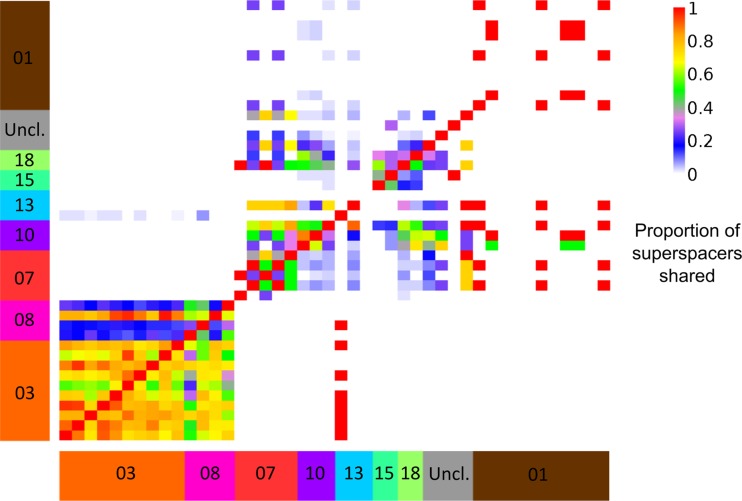
Superspacers shared between viral strains. Each box represents a pair of viruses; the color indicates the proportion of superspacers in the viral strain on the *x* axis which are shared with the virus on the *y* axis. A superspacer is defined as any spacer matching viruses from more than one cluster. Viruses are grouped by cluster (colored boxes to the left and bottom); singletons are grouped together as unclustered (gray box).

Spacer matches are typically not unique to one virus; spacers match 1 to 13 viruses with an average of 2.75 viruses (Fig. S7A). While the number of spacers matching each cluster varies (Fig. S7B), most matched viruses fall within the same cluster. We note that these spacers are useful marker sequences for virus identification and may be used for rapid screening of samples for virus infection. We classify spacers that match more than one virus cluster as “superspacers” and suggest they provide cross-immunity for a single host to multiple viruses (Fig. 4; Table S3). These superspacers are a minority among spacers in our data set; 17% of the spacers matching viruses are considered superspacers. We found only one spacer that matched four nonsingleton virus clusters (clusters 07, 18, 22, and 30), which contain a mix of temperate and lytic viruses. This indicates that some spacers confer immunity to a broad range of viruses. These spacers are not more commonly found in our unique arrays than expected by chance, as would be expected from their broad selective benefit against virus infection (Student’s *t* test, *P* = 0.83 between superspacers and normal spacers found in arrays).

10.1128/mSystems.00075-18.7 FIG S7 Spacer matches to P. aeruginosa viruses. (A) Count of viruses matched per spacer. (B) Number of spacers matching each viral cluster. Download FIG S7, EPS file, 0.4 MB. Copyright © 2018 England et al. 2018 England et al. This content is distributed under the terms of the Creative Commons Attribution 4.0 International license.

10.1128/mSystems.00075-18.10 TABLE S3 Superspacers identified in P. aeruginosa CRISPR arrays. Download Table S3, XLSX file, 0.02 MB. Copyright © 2018 England et al. 2018 England et al. This content is distributed under the terms of the Creative Commons Attribution 4.0 International license.

To determine the frequency of targeting of virus clusters, we quantified the mean number of protospacers per virus, normalized by viral genome size, across all viruses in each cluster (Fig. 3). While most clusters have few protospacers per base pair, five clusters (clusters 3, 8, 10, 14, and 18) and two singletons (22 and 29) exhibited higher targeting (median, >0.001 protospacers per base pair; Fig. 3). Using protospacers per base pair as a measure of targeting, we found two clusters were significantly more frequently targeted than others. Clusters 03 and 08 were targeted significantly more often than 14 and 12 of 18 clusters with at least 3 members, respectively (one-way ANOVAs with Games-Howell test, *P* < 0.05). This breadth of targeting is not primarily due to individual spacers targeting multiple clusters, as evidenced by the small number of superspacers observed (Fig. 4; Table S3).

### Distributed immunity.

We previously observed that distributed immunity, or CRISPR immunity to the same virus via different spacers, has a marked effect on the evolutionary dynamics of host and virus (32). Highly distributed immunity is correlated with increased host population size and composition stability, as well as decreased viral population size and increased viral extinction (32). We quantify distributed immunity within a host, or individual distributed immunity (IDI), as the number of spacer-protospacer matches between a host and a virus, and distributed immunity among hosts, or population distributed immunity (PDI), as incidences of nonshared spacers in two hosts providing immunity to the same virus (see Materials and Methods).

**FIG 5:**
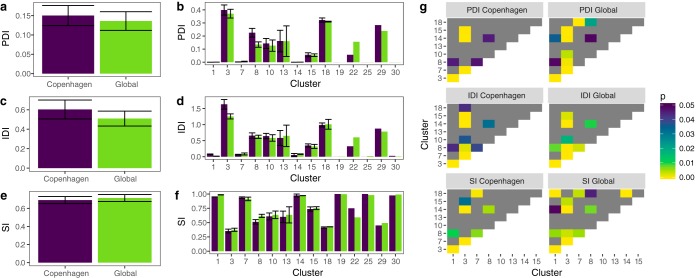
Copenhagen CF strains exhibit higher distributed immunity than the global community. (a) Mean per-virus PDI in host-host-virus trios where both hosts are in the Copenhagen data set (Copenhagen) or outside the Copenhagen data set (Global). PDI: for each pair of hosts, if each host has a spacer matching the virus which is not present in the other host, PDI is 1; else, 0. The mean is taken across all host pairs. (b) Mean PDI by virus cluster targeted. (c) Mean per-virus IDI in Copenhagen versus global strains. IDI, mean number of spacers per host matching a virus. (d) Mean IDI by virus cluster targeted. (e) Susceptibility index (SI) within and outside the Copenhagen data set. SI, count of host-virus pairs where the host is susceptible divided by total host-virus pairs. (f) SI per virus cluster. (g) Heat map of *P* values for significant differences in PDI, IDI, and SI between clusters (one-way ANOVA with Games-Howell test, *P* < 0.05). Gray denotes nonsignificant comparisons.

We observe variation in distributed immunity that correlates with the number of spacers matching each virus. This is consistent with a highly nonoverlapping spacer set from a diversity of CRISPR arrays. We observe higher levels of PDI where both hosts are from the Copenhagen data set than when one or both hosts are outside the local set (Fig. 5a). Similarly, IDI is higher for local strains than for strains from the global population (Fig. 5c). This may reflect repeated sampling of viruses by these CF strains, perhaps due to long-term coexistence in lung environments. Levels of PDI and IDI vary among viral clusters, with clusters 03, 08, 10, 13, 15, and 18 and singletons 22 and 29 in particular exhibiting higher levels of both, whereas other clusters show little evidence of distributed targeting (Fig. 5b and d). The most highly targeted clusters (clusters 03, 08, and 18) are among those with high PDI and IDI, further indicating that these virus types are broadly targeted by P. aeruginosa CRISPRs on a global scale.

Viruses that face high distributed immunity have few susceptible hosts and may evolve differently than those that face lower distributed immunity. This indicates that each virus will have limited susceptible hosts within our global population. To quantify this, we calculated the susceptibility index (SI), or proportion of hosts that lack CRISPR immunity, for each virus (Fig. 5e and f). This metric is consistent with our previous metric for calculating susceptible hosts (HVI) (32); however, HVI requires host and viral relative abundances and is therefore not appropriate for this data set. We find that clusters with high PDI and IDI have correspondingly low SI values, with 37% to 70% of host strains susceptible to these clusters, while clusters with limited distributed immunity retain the ability to infect the vast majority of hosts (Fig. 5f).

## DISCUSSION

Here, we have identified a large, diverse pool of P. aeruginosa CRISPR spacer sequences in both a small, highly sampled CF patient population and a broad sampling of the global P. aeruginosa population. Diversity in the Copenhagen samples reflects that of the global population. Change of CRISPR arrays within a patient over time is limited; however, we observe divergence and recombination of CRISPRs in global data. Comparing these spacers to known P. aeruginosa viruses reveals differential targeting of related viral groups by CRISPRs, with distributed immunity to highly targeted viruses emerging in the global population.

The data sets we incorporated also impose limitations on this study. The Copenhagen data set, while extensive in number of participants and time span, uses genomes from isolates from clinical samples. The number of isolates per sample is small, with fewer than two strains isolated per sample on average. These limited isolations are unlikely to capture the true diversity of P. aeruginosa in these patients and may have limited our ability to capture within-patient CRISPR evolution; however, we were still able to identify numerous strains with related CRISPRs across patients. Publicly available P. aeruginosa genomes present the issue of misassemblies. As these genomes were largely assembled without specific focus on CRISPR regions, missing or misordered spacers are possible due to the repetitive nature of CRISPR arrays, and in most cases sequence reads are not available to facilitate the careful assembly of CRISPR regions used on the Copenhagen data. Despite these possibilities, we still find numerous CRISPR arrays identical to Copenhagen arrays in non-Copenhagen strains (see Table S1 and Fig. S2 in the supplemental material), indicating that there are accurate CRISPR assemblies in this data set. Even if potentially imperfect, these genomes still serve as a valuable source of CRISPR spacers for comparison with Copenhagen and virus data.

Our data clearly show that differential targeting of viruses is divided along the lines of viral lifestyle, with temperate viruses targeted more frequently than lytic viruses. This skewed targeting could indicate that CRISPR immunity is used less frequently for defense against lytic viruses, with other methods being preferred. The most highly targeted cluster was cluster 03; its individual viruses have approximately five protospacers per kb of genome. This cluster contains D3112 and related temperate transposable viruses, whose mosaic genomes have been heavily shaped by horizontal gene transfer among Mu-like and lambda-like viruses (26). These viruses use type IV pili as their receptors (33, 34); these pili are important for motility on solid surfaces and in viscous environments, and for biofilm structure (35). In culture, virus-resistant P. aeruginosa mutants delete the pilus to prevent viral attachment; however, in resistant strains which retain the pilus, CRISPR spacers are added to confer immunity (20). We hypothesize that selective pressure to maintain biofilm structure in environments such as the CF lung prevents strains from gaining resistance via pilus deletion, leading to heavy CRISPR targeting of these viruses. Consistent with this hypothesis, viruses in highly targeted cluster 08 also use pili, including type IV, for entry, as do clusters 14 and 15, which have high to moderate numbers of protospacers (Fig. 3). Some members of highly targeted cluster 03 also possess anti-CRISPRs; though these spacers would be ineffective with an anti-CRISPR system, higher targeting of these strains may result from repeated encounters with these viruses. With no selection against spacers matching viruses integrated into host genomes, matching spacers can remain in host repeat-spacer arrays even if these largely temperate viruses integrate.

There are multiple ecoevolutionary and molecular mechanisms that could result in differences in virus targeting: for example, variable virus abundance, differential selection that results in spacers matching highly targeted viruses being selected for and/or spacers matching infrequently targeted viruses being lost, or differences in virus-host interaction mechanisms that lead to variation in spacer acquisition. With the current data, it is difficult to distinguish among these hypotheses, without abundance of viral clusters in the environments from which these host strains originate. We also lack knowledge of the *R*_0_ of these viruses, making it difficult to predict the dynamics of their invasion and persistence in microbial populations.

Theory predicts that in environments with few susceptible hosts, viruses will tilt their symbiosis toward mutualistic or prudent use of host resources (36–38); this may alter the evolution of interaction traits in these viruses and their impact on host strains. In contrast to the highly diversified global population, *Pseudomonas* viruses within an isolated “island” (39, 40) lung environment face monoclonality of CRISPRs, allowing single-mutation evasion mutants successful access to local hosts. This is similar to the arms race dynamics (41) of selective sweeps associated with surface mutations. In contrast, outside the lung, viruses face distributed immunity and limited host susceptibility. Under these conditions, virus lifestyles may shift toward “rapacious” lifestyles where rapid production of infectious particles is advantageous (36, 42).

In the metapopulation we have described, viruses infecting P. aeruginosa face both immune structures, so the proportion of replication and evolution occurring in each environment will ultimately influence viral phenotypes. It is possible that the diversity we see represents diversity enriched from local source environments; however, the limited change in CRISPR immunity within a patient suggests this is not the case. Instead, we suggest that P. aeruginosa and its viruses migrate, interact, and evolve between environments. Further characterization of viral diversity is needed to fully elucidate the structure of diversity reflected in CRISPRs.

Distributed immunity would be expected to limit the spread of proviruses, as CRISPR targeting of an integrated provirus would result in degradation of the host genome. While distributed immunity can depress the spread of previously encountered viruses, it also creates an opportunity for phages with dissimilar genomes to invade a population, as they would be subject to less CRISPR targeting and have less competition for hosts. Such gaps in antiviral defense could be exploited for virus therapy; by carefully selecting lytic viruses with minimal similarity to common viral genomes, one could limit the ability of CRISPRs to interfere with the therapeutic phage. An example from this study is cluster 06; these phages are mostly lytic and have low similarity and few shared protospacers with other clusters (Fig. 2 and Fig. S6A and B). In addition, it may be possible to immunize *Pseudomonas* strains against viruses that increase virulence and pathogenicity to limit the spread of these phenotypes.

These results depict a global population of P. aeruginosa and viruses where many virus types are circulating across a broad geographic area in multiple environments. Host CRISPRs bear evidence of encounters with many types of viruses without an environmental pattern. The increased targeting of certain largely temperate virus groups suggests that hosts have various immune responses to different virus types. Using a large library of spacers extracted from an extensive data set spread across time, space, and sample type allowed us to see how these viruses were differentially targeted on a global scale. Applying this type of surveillance to other host-virus systems could similarly reveal novel patterns in CRISPR targeting and viral population structure.

## MATERIALS AND METHODS

### Host data set selection.

The set of P. aeruginosa strains analyzed in this paper includes data from several sources. Reads associated with 458 P. aeruginosa strains cultured from patient samples collected from the Copenhagen Cystic Fibrosis Center at the University Hospital, Rigshospitalet, Denmark (24), were retrieved from the NCBI Sequence Read Archive (accession no. ERP004853). Assembled genomes of 24 P. aeruginosa strains described in reference 43 were kindly provided by the authors (GenBank accession no. AWYJ00000000 to AWZG00000000). Assembled genomes of 388 strains described in reference 44 were obtained from GenBank (BioProject accession no. PRJNA264310). All other complete and draft-stage P. aeruginosa genomes were retrieved from the NCBI Nucleotide database in September 2014 (310 genomes; accession numbers in Table S1). CRISPR arrays from reference 19 were downloaded from NCBI (45 sequences). Three additional sets of CRISPR arrays were obtained from metagenomic sequence of three CF sputum samples kindly provided by Katrine Whiteson and Yan Wei Lim. Metadata including isolation location, sampling date, environment, and epidemic strain status were collected where possible (Table S1).

### Quality filtering and genome assembly.

For all samples with sequencing reads available, reads were trimmed and quality filtered using Prinseq 0.20.4 (45). Reads were trimmed from both ends using a 5-nt sliding window with a minimum quality score of 30. Reads were retained if they had a mean quality score of 30 and <1% ambiguous bases. The minimum read length was set to two-thirds the anticipated read length, or 66 nt. Draft assemblies were generated with MIRA 4.0 (46) using genome, *de novo*, and accurate parameters.

### CRISPR identification and spacer extraction.

CRISPR arrays were identified via BLASTn of known P. aeruginosa CRISPR repeats (19). Parameters were adjusted for short search sequence and to maximize hits covering the entire repeat length as follows: “-word _size 7 -gapopen 3 -gapextend 2 -reward 1 -penalty -1.” The minimum percent identity was set to 80 to allow for degenerate repeat sequences. Hits <24 bp were filtered from the results. Sequences with a repeat of the same type both up- and downstream in the same orientation and <40 bp away from other hits were considered spacers and extracted. A spacer rarefaction curve was computed in QIIME (47).

CRISPR array ranges were declared as all consecutive repeats and spacers in the same orientation <500 bp away from one another. Groups of repeats and spacers on different contigs, on the same contig/genome in different orientations, or on the same contig/genome but separated by >500 bp were considered separate arrays.

For samples with reads available, CRISPR arrays were further verified for accuracy and completeness using a technique called nonassembled repeat boundary linkage, or NARBL (http://github.com/englandwe/NARBL). To establish spacer order, repeats were identified on sequence reads, and 12-nucleotide “chunks” of DNA flanking each repeat were identified using fuzznuc (48); up to 8 mismatches to the repeat sequence were permitted. When the repeat was matched in both orientations due to palindromic repeats, the match with fewer mismatches was kept. Chunks that were a perfect match to the repeat sequence (i.e., from adjacent or partial repeats) were also discarded. Finally, singleton chunks that perfectly overlap nonsingleton chunks by at least 8 bp were removed, to account for rare chunks generated by sequencing error.

Occurrences of two or more chunks on the same read were recorded as links, which represent either two ends of the same spacer or opposite ends of two spacers linked across a repeat. The first type was used to identify spacer sequences; the second, to order spacers. Linkage networks were analyzed using Cytoscape (49). Based on average repeat and spacer lengths of species with previously sequenced CRISPR arrays, links spanning a single repeat-spacer unit were considered short links, spanning only a single spacer or pair of adjacent spacers; longer links were considered to span multiple spacers and were not counted when determining coverage of links. All spacer sequences used in this study can be found in Table S1.

### Multilocus sequence typing.

An established panel of seven markers (25) was used for MLST analysis. MLST loci were identified by BLASTn (50) of a representative known allele obtained from the Pseudomonas aeruginosa PubMLST website (http://pubmlst.org/paeruginosa/) (51) against genomes or contigs. The best BLAST hit for each MLST locus was then BLASTed against a database of all known alleles for that locus, also from the PubMLST website. Exact matches to a known allele were assigned that allele’s ID number; hits with lower identity or incomplete coverage of the locus were investigated manually, and any identified as novel alleles were assigned new ID numbers of >10,000. Strains with inconclusive MLST alleles were removed from further MLST analysis. A maximum-likelihood tree of concatenated MLST markers was constructed with RAxML (52) using the rapid bootstrapping algorithm plus maximum likelihood and GTRgamma nucleotide substitution model with 100 bootstrap replicates.

### Virus data set selection and protospacer identification.

Genomes of all viruses identified as infecting P. aeruginosa were downloaded from the NCBI Nucleotide database on June 23, 2015, totaling 92 unique viruses. Six previously identified proviruses from P. aeruginosa LESB58 were added using genomic coordinates from reference 12. All viruses were classified according to lifestyle (lytic, temperate, nonlytic, or unknown) based on literature descriptions. These 98 viruses and proviruses were used for all virus-related analyses.

Protospacers in virus genomes were identified via BLASTn of spacer sequences. The parameter “-task blastn-short” was used due to short query length. A minimum E value of 0.01 was used to capture incomplete and imperfect matches, allowing up to four mismatches over a full-length match. PAMs were identified and partial-length matches were extended to cover the full spacer length using clDB (53), and the Hamming distance between protospacer and spacer was calculated. Protospacer matches were kept if a correct PAM sequence was present. Acceptable PAM sequences were GG or TTC, indicative of type 1-F and type 1-E PAMs, respectively. Any matches with a Hamming distance of >3 were filtered out of analysis. Spacers matching protospacers on more than one distinct cluster were designated “superspacers.”

### Assignment of viruses to genome clusters.

To assign viruses to clusters, all virus genomes were compared using BLASTn (E < 0.001). For each pair of genomes, the proportional length alignment (PLA), or total length aligned by BLAST over the length of the query, was calculated and used as our measure of viral similarity. MCL (54) was used to cluster viruses into networks with edges weighted by PLA with a minimum PLA cutoff of 0.2.

### Distributed immunity and susceptibility index.

Population distributed immunity (PDI) was calculated on a per-virus basis using all possible pairs of hosts. For each host-host pair, if each host has a spacer matching the virus which is not present in the other host, PDI is 1; else, PDI is 0. At the population level, PDI is then averaged across all host-host pairs. Criteria for matching spacers and protospacers are as described above. Individual distributed immunity (IDI) is measured as a count of spacers in a host matching a virus. At the population level, IDI is averaged across all hosts. The susceptibility index (SI) is the number of host-virus pairs where the host is not immune to the virus divided by the total number of host-virus pairs. Immunity is defined as a spacer-protospacer match as described above.

### Statistics.

All statistical tests were performed in R versions 3.2.2 to 3.2.4 (55). Games-Howell tests were performed using the userfriendlyscience package (56). Plots were generated in R using the ggplot2 package (57).
