# Supplementary figures and images for: Metapopulation Structure of CRISPR-Cas Immunity in Pseudomonas aeruginosa and Its Viruses
Source: mSystems. 2018 Oct 23;3(5):e00075-18. doi: 10.1128/mSystems.00075-18 (PMC6199469; doi:10.1128/mSystems.00075-18)

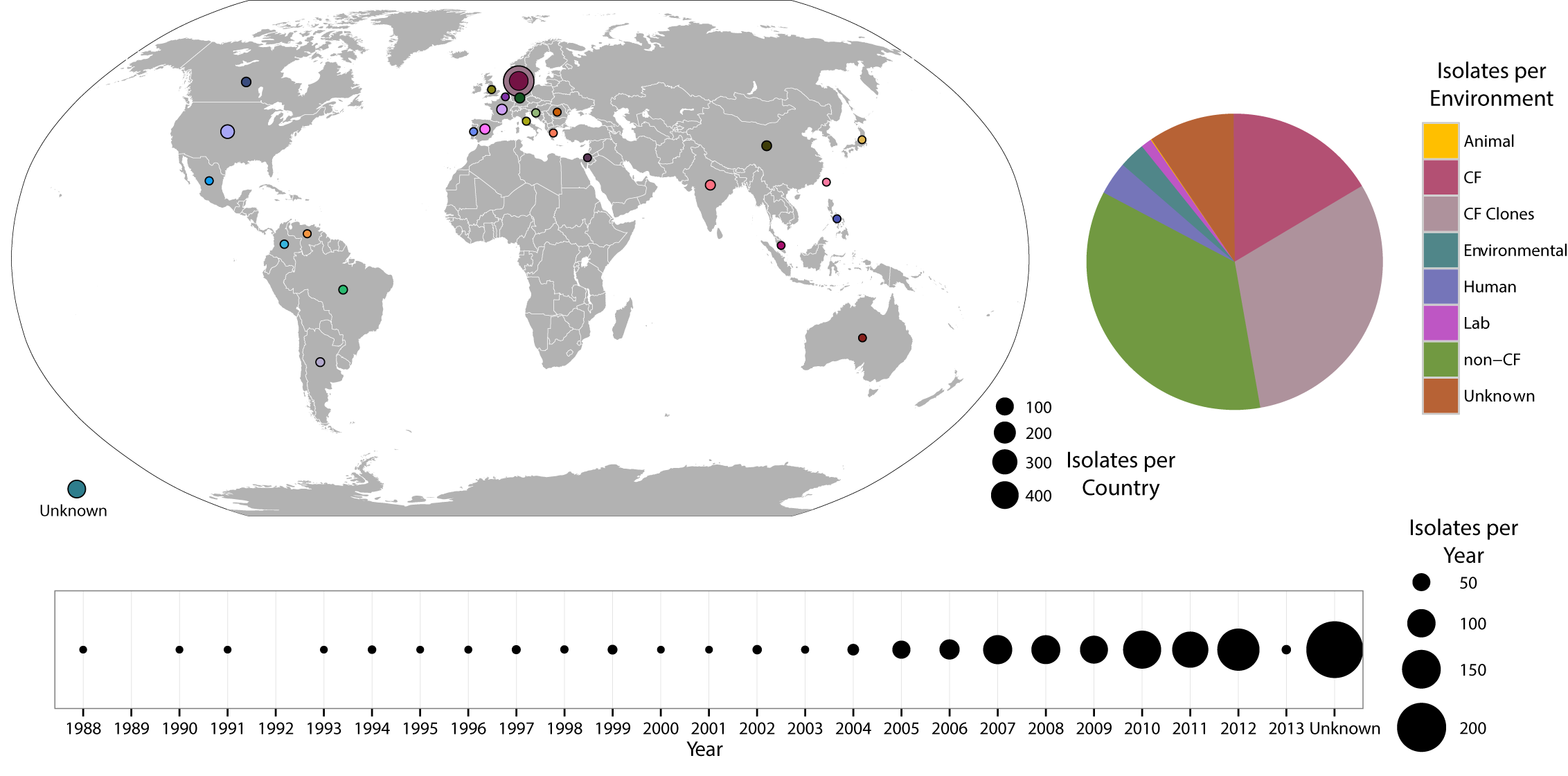

Supplement: FIG S3 [file sys005182276sf3.tif]
